# A dual methane production mechanism mediated by enzymatic catalysis and the Fenton reaction in the wood-rotting fungus *Schizophyllum commune*

**DOI:** 10.1128/aem.01298-26

**Published:** 2026-08-27

**Authors:** Mengshi Zhao, Yu Xiao, Shuang Leng, Jiasong Fang, Changhong Liu

**Affiliations:** 1 State Key Laboratory of Pharmaceutical Biotechnology, Nanjing University 384750, Nanjing, China; 2 Shanghai Engineering Research Center of Hadal Science and Technology, College of Marine Sciences, Shanghai Ocean University 74595, Shanghai, China; 3 Laboratory for Marine Biology and Biotechnology, Qingdao Marine Science and Technology Center, Qingdao, China; University of Delaware, Lewes, Delaware, USA

**Keywords:** *Schizophyllum commune*, fungal methanogenesis, CRISPR-Cas9, dual enzymatic-Fenton mechanism, environmental factors, reactive oxygen species

## Abstract

Understanding biological sources of methane is critical for elucidating the global methane cycle and addressing climate change. While traditional research has predominantly focused on prokaryotic methanogens, emerging evidence suggests that eukaryotes, particularly fungi, can produce methane through both enzymatic and non-enzymatic pathways. However, no eukaryotic organism has been documented to possess both pathways simultaneously. Here, we provide preliminary evidence suggesting that the wood-decaying fungus *Schizophyllum commune* strain 20R-7-F01, isolated from sediment approximately 2 km below the seafloor, employs dual mechanisms for methane production. By comparing wild-type strains with mct1 (methyl chloride transferase 1) and dh3 (dehalogenase 3) deficient strains under iron-replete (50 μM Fe³^+^, to facilitate Fenton reactions) and iron-free conditions, we estimated that enzymatic pathways account for roughly 82%–85% of total methane production. The remaining ~15%–18% of methane output is consistent with a contribution from Fenton-type chemistry, and this quantitative partitioning of pathway contributions was derived from cross-condition yield comparisons. Environmental factors such as oxygen, temperature, and substrate type significantly influence enzymatic efficiency, whereas the efficacy of the non-enzymatic pathway depends on the concentration of methyl donors, particularly dimethyl sulfoxide. Additionally, both pathways are activated by hydrogen peroxide, indicating that reactive oxygen species positively regulate methane synthesis. This study reveals a novel co-existing “enzymatic-Fenton” mechanism for methane production in fungi, highlighting their crucial role in the global methane budget and carbon cycling.

**IMPORTANCE:**

Methane is a key driver of climate change, yet our understanding of its biological sources has long been limited to bacteria and archaea. Here, we reveal that a fungus, *Schizophyllum commune* 20R-7-F01, isolated from deep below the seafloor, produces methane through two distinct mechanisms operating simultaneously: an enzymatic pathway and a Fenton reaction-driven non-enzymatic pathway. This dual system, previously unreported in any eukaryote, redefines the potential contribution of fungi to the global methane budget. Our findings challenge the traditional prokaryote-centric view of methanogenesis and open new avenues for understanding methane cycling in oxygen-rich and iron-rich environments, with implications for climate modeling and carbon cycle research.

## INTRODUCTION

Methane (CH₄) is a potent greenhouse gas whose global warming potential far exceeds that of carbon dioxide (CO₂), making it a critical driver of climate change dynamics (1). Understanding the biological origins of methane is therefore essential for devising effective mitigation strategies (2). Historically, research on biogenic methane has focused on prokaryotic methanogens as the principal producers. However, recent work has demonstrated that several bacterial lineages also possess methanogenic capacity (3, 4).

Beyond these prokaryotic sources, accumulating evidence indicates that fungi can generate methane through two mechanistically distinct routes: (i) an enzyme-mediated pathway involving methyltransferases and dehalogenases and (ii) an abiotic, reactive oxygen species (ROS)-driven Fenton reaction in which ferrous iron (Fe²^+^) reacts with ROS to liberate methane under aerobic conditions (5–7). The Fenton pathway has been implicated in prebiotic chemistry on the early Earth, presumably because Fe²^+^ and methylated substrates were abundant in ancient environments (8).

*Schizophyllum commune*, a wood-decaying basidiomycete, has emerged as a tractable model for probing fungal methanogenesis. Genome annotation revealed two methyltransferase genes (*mct1* and *mct2*) and five dehalogenase genes (*dh1–dh5*). RT-qPCR showed that transcription of *mct1*, *dh3*, *dh4*, and *dh5* is strongly induced when the fungus is cultivated on methanogenic substrates such as glucose, lignin, phenol, and p-anisic acid, whereas expression remains low on non-methanogenic carbon sources (methanol, benzene). Notably, only *dh3* is significantly upregulated when chloromethane (CH₃Cl) serves as the sole carbon source, implicating the mct1-encoded methyl chloride transferase and the *dh3*-encoded dehalogenase as core components of an anaerobic CH₄-producing pathway (9). Consistent with this, methane evolution by *S. commune* is markedly enhanced under high hydrostatic pressure (HHP), approximately 2.5 times higher levels under 35 MPa compared to 0.1 MPa (standard atmospheric pressure), a condition that elevates intracellular ROS levels (10). Preliminary observations also suggest that the fungus can employ a Fenton-type reaction during aerobic lignocellulose degradation (11–13).

Despite these advances, no study has experimentally verified that a single eukaryotic organism can simultaneously operate both enzyme-mediated and Fenton-based methane production pathways under ecologically relevant conditions. Moreover, the influence of environmental variables on the activity and relative contributions of these two routes, as well as their broader ecological significance, remains poorly characterized.

To fill these gaps, we selected *S. commune* strain 20R-7-F01, which retains the putative methane-synthesis enzymes mct and dh, as our model organism. We hypothesized that methane production in this strain proceeds via two parallel pathways: (i) an enzyme-mediated route driven by mct and dh activities and (ii) an abiotic Fenton-mediated route driven by ROS-generated Fe²^+^. Classical homogeneous Fenton chemistry relies on free Fe²^+^ as the catalytically active species; however, direct Fe²^+^ supplementation is unsuitable for fungal cultures because Fe²^+^ is rapidly oxidized to Fe³^+^ in oxic, metabolically active media, and high concentrations provoke non-specific oxidative damage that impairs fungal physiology (14). In natural wood-decay fungi such as *S. commune*, Fe³⁺ is typically encountered as the predominant iron species in their habitats, which can be gradually reduced to Fe²^+^ by fungal-secreted reductive metabolites (e.g., organic acids and extracellular reductases). This endogenous reduction sustains a mild, prolonged Fenton reaction that aligns with fungal metabolic timescales without incurring acute cytotoxicity (15).

Guided by this rationale, we hypothesize that *S. commune* employs both enzymatic and Fenton-mediated methanogenesis pathways. By constructing *mct1* and *dh3* knockout mutants and comparing their methane production with the wild type under varying Fe^3+^, temperature, and substrate conditions, we aim to demonstrate pathway coexistence and quantify their relative contributions. Elucidating these mechanisms will advance understanding of fungal methanogenesis, clarify fungal impacts on the global methane budget, and illuminate their role in carbon cycling.

## MATERIALS AND METHODS

### Construction of mutant strains deficient in enzymatic methane-production genes

The methyltransferase gene *mct1* and dehalogenase gene *dh3* of *S. commune* strain 20R-7-F01 were targeted for deletion using a CRISPR-Cas9 system to generate mutant strains lacking genes involved in enzymatic methane production. First, single-guide RNAs (sgRNAs) specific to each open reading frame (ORF) were designed using Benchling and cloned into pCRISPR-Cas9 vectors carrying either a hygromycin B or bleomycin resistance cassette (Table S1). Protoplasts of the wild-type strain were then transformed with the corresponding plasmids, and transformants were selected on media supplemented with hygromycin B (for *mct1* edits) or bleomycin (for *dh3* edits). Additionally, a double mutant (Δmct1-dh3) was generated. Complementary strains (Δmct1/pmct1 and Δdh3/pdh3) were also constructed (16, 17).

Knockout of the target genes was verified by genomic PCR across the target loci followed by Sanger sequencing to confirm precise deletions, and residual transcript levels of the targeted genes were quantified by RT-qPCR [detailed in “Real-time quantitative PCR (RT-qPCR),” below], which showed levels of less than 5% of the wild-type control (Table S2; Fig. S1). The final strain library included single mutants Δmct1 and Δdh3; the double mutant Δmct1-dh3; and complementary strains Δmct1/pmct1 and Δdh3/pdh3, in which the wild-type coding sequences were reintroduced at their native loci. All strains were stored as glycerol stocks at −80°C and routinely maintained on potato dextrose agar (PDA) at 25°C.

### Culture medium and growth conditions

An artificial iron-free seawater medium (per liter: 27 g NaCl, 5 g MgCl₂·6H₂O, 0.7 g KCl, 0.2 g CaCl₂·2H₂O, 0.2 g NaHCO₃, trace vitamin mix, pH 7.8) was prepared using Chelex-100-treated water to eliminate contaminating metals, with two iron treatment regimens established: the iron-supplemented (+Fe) group was amended with ferric chloride to a final concentration of 50 µM, while the iron-free (–Fe) group contained no added FeCl₃, and all reagents were passed through Chelex resin to ensure complete iron depletion (18). Cultures were inoculated with 7 g wet weight of mycelium into glass bottles containing 170 mL of medium, sealed with rubber septa, and incubated anaerobically (N₂-flushed) at 30°C unless otherwise stated, whereas aerobic control flasks were left open to ambient air (~21% O₂). The following experimental variables were each tested in triplicate: oxygen levels (0% anaerobic vs 21% aerobic), temperature (30°C vs 40°C), H₂O₂ concentrations (0, 0.75, 1.5, and 3 mM), and carbon sources (glucose, galactose, o-anisic acid, phenol, and DMSO, all supplied at 10 mM). Mycelia were harvested at 1, 3, and 5 days post-inoculation, and headspace gas was sampled at the same time points for methane analysis.

### Mycelial biomass and viability

Harvested mycelia were filtered through pre-weighed glass-fiber filters, rinsed with sterile deionized water, blotted dry, and either dried at 60°C to constant weight for dry weight (DW) recording or weighed immediately post-filtration to obtain wet weight (WW) for normalization of methane yields (nmol CH₄ /mg WW) (10). For viability assessment, aliquots of fresh mycelia were stained with 0.4% trypan blue for 5 min, with viable hyphae remaining colorless and non-viable hyphae turning blue, and viability (%) was calculated as (colorless hyphae / total hyphae) × 100 (19, 20).

### Methane and ROS detection

Headspace gas (1 mL) was withdrawn with a gas-tight syringe and injected into an Agilent 7890 GC equipped with a flame-ionization detector (FID). Conditions were as follows: N₂ carrier gas (1.3 mL min⁻¹), HP-PLOT/Q column (3.2 m  ×  1/8 in, 80/100 mesh), oven temperature of 40°C, and FID temperature of 300°C. Calibration standards (0.1–5 ppmv CH₄ in N₂) were run in triplicate. A linear regression (*R*²  > 0.998) converted peak area to concentration. Total methane (nmol) = [CH₄]  ×  headspace vol (determined by water displacement) (21). Heat-killed controls (autoclaved 121°C, 20 min) yielded CH₄  <  0.01  nmol/ mg, confirming the biological origin.

Intracellular ROS were measured with the DCFH-DA assay (22, 23). Fungal mycelia were harvested after 1, 3, and 5 days of incubation. For ROS measurement, mycelial samples (≈10 mg WW) were incubated with 10 µM DCFH-DA in PBS (pH 7.4) for 30 min at 30°C, washed twice, and fluorescence (Ex 485 nm/Em 535 nm) recorded on a plate reader (Fig. S2). Results were expressed as relative fluorescence units (AU) per mg WW.

### Quantification of enzymatic vs Fenton-like contributions

To quantify the relative contributions of the enzymatic mct/dh pathway and the Fe-driven Fenton-like pathway to total methane production, we first validated the additivity of the two pathways (i.e., total methane yield equals the sum of individual pathway outputs with no synergistic or inhibitory interactions) prior to formal partitioning. Four experimental groups were set for additivity validation: wild type (WT) under iron-replete (+Fe) conditions (total methane yield designated as A), WT under iron-free (–Fe) conditions (yield from enzymatic pathway only, designated as B), Δmct1-dh3 mutant under +Fe conditions (enzymatic pathway blocked, yield from Fenton-like pathway only, designated as C), and Δmct1-dh3 mutant under –Fe conditions (negative control, expected to produce no significant methane, designated as D). The percentage contribution of each pathway was calculated using the following formulas:


$$\begin{array}{rl} & \text{ Enzymatic contribution }(\%)=\frac{{CH}_{4}^{WT-Fe}}{{CH}_{4}^{WT+Fe}}\times 100\\ & \text{ Fenton-like contribution }(\%)=\frac{{CH}_{4}^{\Delta mct1\text{-}dh3+Fe}}{{CH}_{4}^{WT+Fe}}\times 100 \end{array}$$

where CH_4_^WT+Fe^ denotes the methane yield of WT under iron-supplemented conditions (normalized to 100%); CH_4_^WT−Fe^ denotes the methane yield of WT under iron-free conditions; and CH_4_^Δmct1-dh3+Fe^ denotes the methane yield of the Δmct1-dh3 mutant under iron-supplemented conditions. Additivity was validated using a paired Student’s *t*-test comparing the observed total WT yield (A) with the theoretical summed value of B and C.

### Real-time quantitative PCR (RT-qPCR)

Total RNA was extracted from mycelia using TRIzol reagent, treated with DNase I, and reverse-transcribed with random hexamers. RT-qPCR was performed on a Bio-Rad CFX96 system using ChamQ Universal SYBR qPCR Master Mix. β-actin served as the internal reference. Relative expression was calculated by the 2^−ΔΔCt^ method with each sample run in technical triplicate and biological triplicate (*n*  =  3) (24).

### Statistical analysis

All data are presented as mean  ±  standard deviation (SD) of three independent biological replicates. For multiple-group comparisons, one-way ANOVA followed by Tukey’s post-hoc test was used. For pairwise comparisons, two-tailed unpaired Student’s *t*-test was applied; Pearson’s correlation coefficient (*r*) was calculated between ROS levels and methane production. Statistical significance was accepted at *P*  <  0.05 (25). All analyses were performed with GraphPad Prism 8.0.2, and correlation plots were generated in Origin 8.5.

## RESULTS AND DISCUSSION

### Key genes in methane synthesis of *S. commune*

Previous studies have shown that *S. commune* 20R-7-F01 can produce methane under anaerobic conditions, with mct1 and dh3 identified as key enzymes involved in this process (9). To further verify the biological functions of *mct1* and *dh3*, we generated corresponding gene knockout strains using CRISPR-Cas9. Single (Δmct1, Δdh3) and double (Δmct1-dh3) deletion mutants were selected based on hygromycin and zeocin resistance. PCR with flanking primers yielded amplicons of identical size to the wild type in all mutants, excluding large genomic rearrangements. Sequencing, however, revealed distinct editing events at each target site: Δmct1 had a 2-bp deletion (−2 G); Δdh3 showed the same (+1 C; −1 T); and Δmct1-dh3 carried a 2-bp insertion, a 1-bp deletion, and a G→A substitution (+2 T; −1 G; G→A). These mutations introduced frameshifts or amino acid changes that disrupted gene function. qPCR confirmed that target gene transcripts were undetectable in all mutants, verifying successful knockout. To exclude off-target effects, we constructed complementary strains Δmct1/pmct1 and Δdh3/pdh3. qPCR showed that *mct1* and *dh3* expression in these strains was restored to wild-type levels, with no significant differences, confirming successful complementation and their suitability for functional rescue assays (Fig. S1).

**Fig 1:**
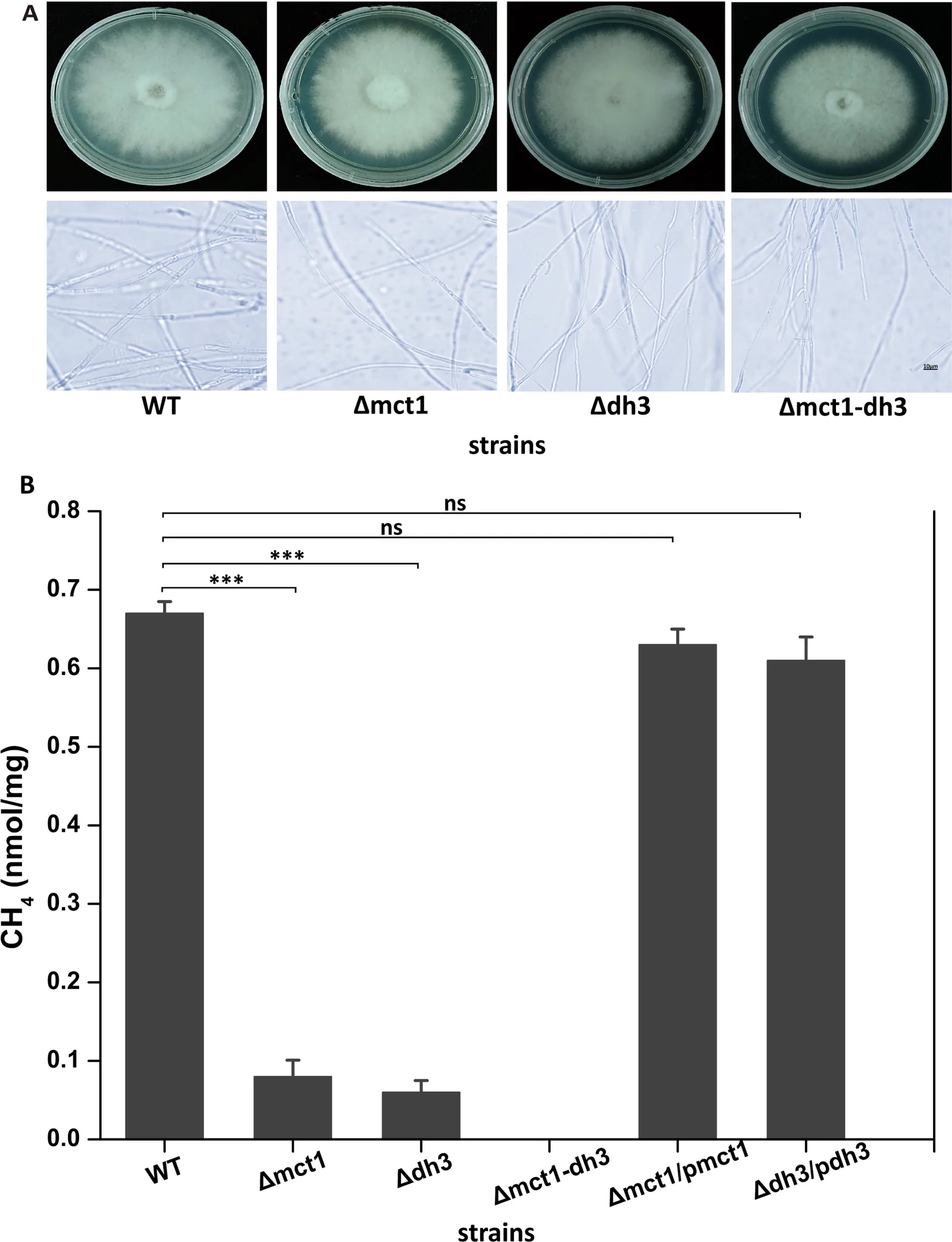
Methane production capacity of gene deletion strains in *S. commune*. Wild-type (WT) and indicated mutant strains were cultivated under anaerobic conditions at 30°C for 3 days. Methane yields are expressed as nmol per mg wet biomass (mean ± SD, *n* = 3 biological replicates). Asterisks indicate significant differences compared to WT as determined by one-way ANOVA with Tukey’s post-hoc test: *P < 0.05, **P < 0.01, ***P < 0.001; ns, not significant (*P* ≥ 0.05).

Our results revealed that the mycelial growth and morphology of all gene deletion strains were comparable to those of the WT strain (Fig. 1A). In contrast, methane production was significantly reduced in the deletion strains: Δmct1 produced 0.08 nmol/mg, Δdh3 yielded 0.06 nmol/mg, and Δmct1-dh3 exhibited no detectable methane, whereas the WT strain produced 0.67 nmol/mg. Complementary strains Δmct1/pmct1 and Δdh3/pdh3 restored methane production to near WT levels (0.61 nmol/mg and 0.65 nmol/mg, respectively). Importantly, no methane was detected in uninoculated culture media, excluding abiotic methane formation and confirming that the measured methane was specifically derived from strain metabolism.

This phenotypic pattern of “unchanged morphology but differentiated functionality” carries multiple biological implications. First, the overall decoupling between morphological and functional phenotypes clearly indicates that the methyltransferase (mct) and dehalogenase (dh) genes are metabolism-specific genes rather than those essential for basal hyphal growth—they are directly involved in enzymatic catalytic steps within the methanogenic pathway rather than in structural processes such as cell wall synthesis or hyphal extension. The normal hyphal growth observed in all knockout strains rules out the interference of global cellular “sickness,” thereby allowing the specific reduction in methanogenic capacity to be cleanly attributed to the direct loss of function of the target genes. Second, the intrinsic diversity of *mct* and *dh* families in fungal genomes underscores their importance in fungal growth and metabolism. The presence of multiple homologous genes constitutes a network of functional redundancy and division of labor, ensuring that fungi maintain normal physiological metabolism under complex and changing environmental conditions. Different homologs may play more prominent roles in specific metabolic branches or environmental contexts. This functional differentiation is well-illustrated by other gene families in sulfur metabolism. In *Saccharomyces cerevisiae*, the sulfite reductase is composed of α and β subunits encoded by distinct genes (*MET10* and *MET5*), demonstrating a molecular-level division of labor (26). Additionally, gene duplication can lead to functional divergence, as seen in the black truffle *Tuber melanosporum*, where two PAPS reductase paralogs have evolved distinct roles: one retains catalytic activity while the other functions as a transcriptional regulator (27). These examples indicate that homologous genes in sulfur assimilation have evolved specialized functions rather than simple redundancy. Similarly, in the present study, deletion of *mct1* and *dh3* resulted in pronounced methanogenic defects, indicating that these two genes serve critical catalytic functions in this pathway. Nevertheless, the residual trace yields (0.06–0.08 nmol/mg) observed in single knockout strains imply partial functional compensation by other family members (9), further supporting the notion of a functionally differentiated and compensatory genetic network. Alternatively, beyond this partial functional compensation hypothesis, this trace residual methane might also function as an intracellular signaling molecule, a hypothesis currently supported by observations that exogenous methane alleviates oxidative stress in diverse model organisms (28–30). Critically, we explicitly note that the above supporting evidence is derived from exogenous methane treatment studies, and the endogenous signaling role of methane in our wood-decay fungal system remains hypothetical pending further experimental validation.

### Coexistence of enzymatic and iron-dependent non-enzymatic methane production pathways in *S. commune*

To quantify the independent contributions of enzymatic and non-enzymatic mechanisms to methane generation in *S. commune* 20R-7-F01, we systematically compared methane yields of the WT and Δmct1-dh3 strains cultivated under iron-free and 50 μM Fe³^+^-supplemented conditions. This experimental framework enabled direct separation of pathway-specific contributions to total methane output.

**Fig 2:**
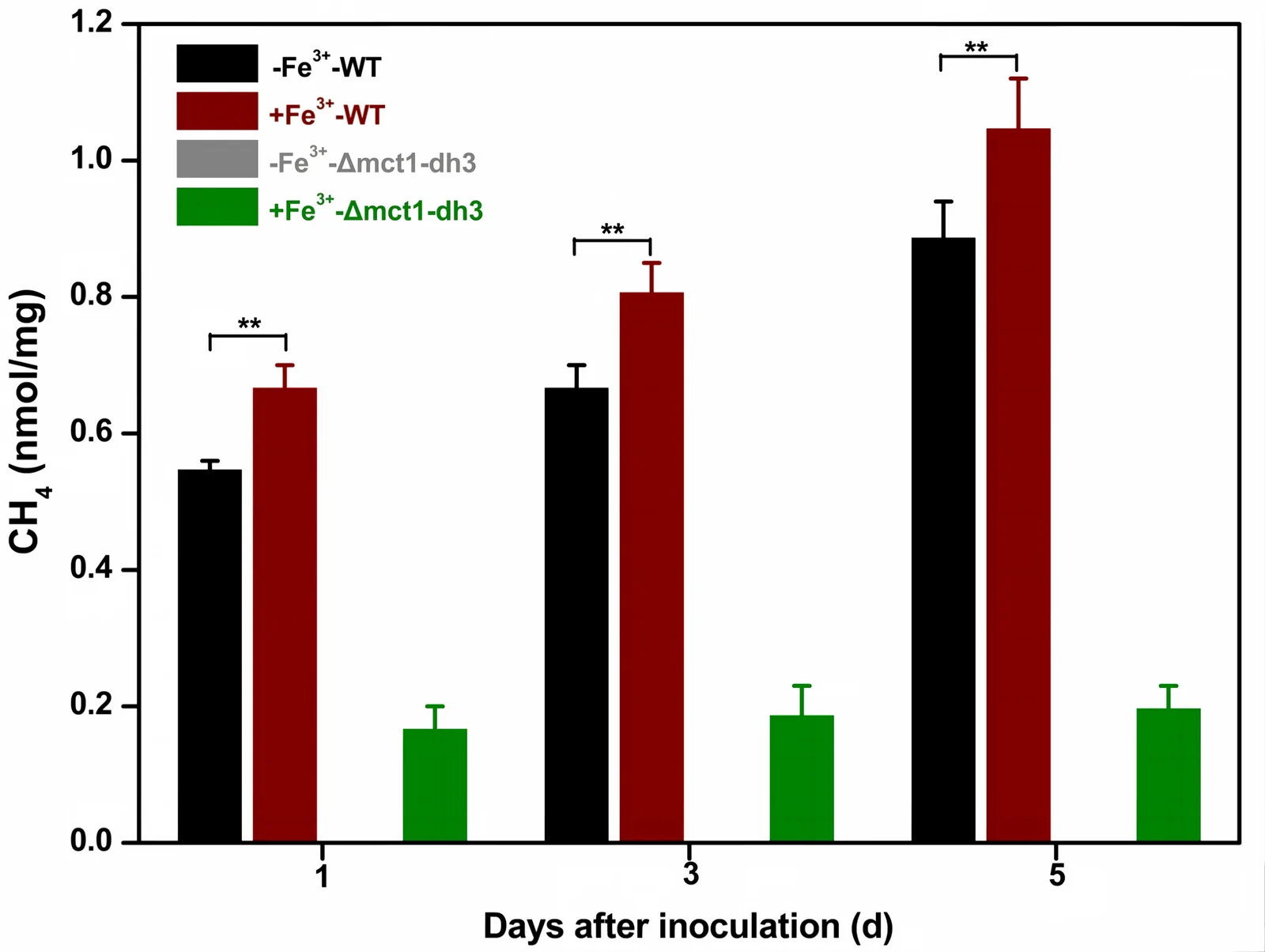
Specific methane production by WT and Δmct1-dh3 strains in iron-containing and iron-free media. *P < 0.05, **P < 0.01, ***P < 0.001; ns, not significant (*P* ≥ 0.05).

Results confirmed that *S. commune* strain 20R-7-F01 relies on both enzymatic and non-enzymatic pathways for methane production. Under iron-free conditions, the WT strain accumulated methane yields of 0.55, 0.67, and 0.89 nmol/mg after 1, 3, and 5 days of cultivation, respectively. In contrast, the Δmct1-dh3 strain produced no detectable methane throughout the entire incubation period, confirming that the enzymatic CH₄ synthesis pathway operates fully independently of exogenous iron availability (Fig. 2).

When exogenous Fe³^+^ was supplied, both the WT and Δmct1-dh3 strains displayed significantly higher methane yields compared with the iron-free conditions, demonstrating the existence of an iron-associated methane generation pathway consistent with Fenton-like redox chemistry. Specifically, methane production by the Δmct1-dh3 strain in iron-rich media remained stable over time, averaging 0.14 nmol/mg biomass, while the WT strain’s methane output increased at comparable rates in both iron-containing (~0.5 nmol/mg/day) and iron-free (~0.6 nmol/mg/day) media (Fig. 2).

Paired *t*-test analysis showed that, at 1, 3, and 5 days of cultivation, there were no significant differences between the observed total methane yield of the WT strain (A) and the summed theoretical value of B and C at all three time points (*P* > 0.05). Furthermore, no significant differences were detected between the inferred non-enzymatic yield (A−B) and the mutant yield C (*P* > 0.05), nor between the inferred enzymatic yield (A−C) and B (*P* > 0.05), indicating the absence of substantial synergistic or inhibitory interactions between the two pathways under the tested conditions (Table S3). Based on the validated additivity, approximately 82–85% of total methane produced by this strain originated from the enzymatic mct/dh pathway, while the remaining 15–18% arose from the iron-dependent Fenton-like process. It should be noted that this estimate is derived from the observed additivity of pathway outputs rather than direct validation of Fenton reaction activity (e.g., hydroxyl radical detection or specific inhibitor assays); therefore, it should be interpreted as an observational estimate rather than a definitive attribution of pathway contribution.

While previous studies have reported Fenton reaction-driven methane production in aerobic fungi such as *Saccharomyces cerevisiae* S288C and *Aspergillus niger* DSM 821 (6), this study provides preliminary evidence documenting a fungus generating methane via a non-enzymatic pathway under anaerobic conditions, a finding that may expand our understanding of fungal contributions to global methane fluxes.

Our results demonstrate that *S. commune* strain 20R-7-F01 harbors coexisting, independent enzymatic and iron-dependent, non-enzymatic methane production pathways. This finding not only advances fundamental knowledge of methane generation mechanisms in eukaryotic organisms but also offers a useful framework for investigating whether similar dual-pathway methane production exists in other ecologically relevant species. Although direct evidence for dual-pathway methane production remains limited in other methane-producing organisms to date, preliminary data support the possibility of analogous mechanisms in some archaea: for example, *Methanococcoides orientis* LMO-1 exhibits significantly elevated methane production in iron-containing media, suggesting that it may also harbor a Fenton reaction-mediated non-enzymatic methane production pathway (31).

### Impact of environmental factors on methane production via enzymatic and non-Enzymatic pathways in *S. commune*

To investigate the effects of oxygen, temperature, substrate, and H₂O₂ concentration on methane production through both enzymatic and non-enzymatic pathways in *S. commune* strain 20R-7-F01, we cultured the WT strain in iron-free media to represent the enzymatic pathway and the Δmct1-dh3 strain in iron-containing medium to represent the iron-dependent non-enzymatic pathway over a 3-day incubation period.

**Fig 3:**
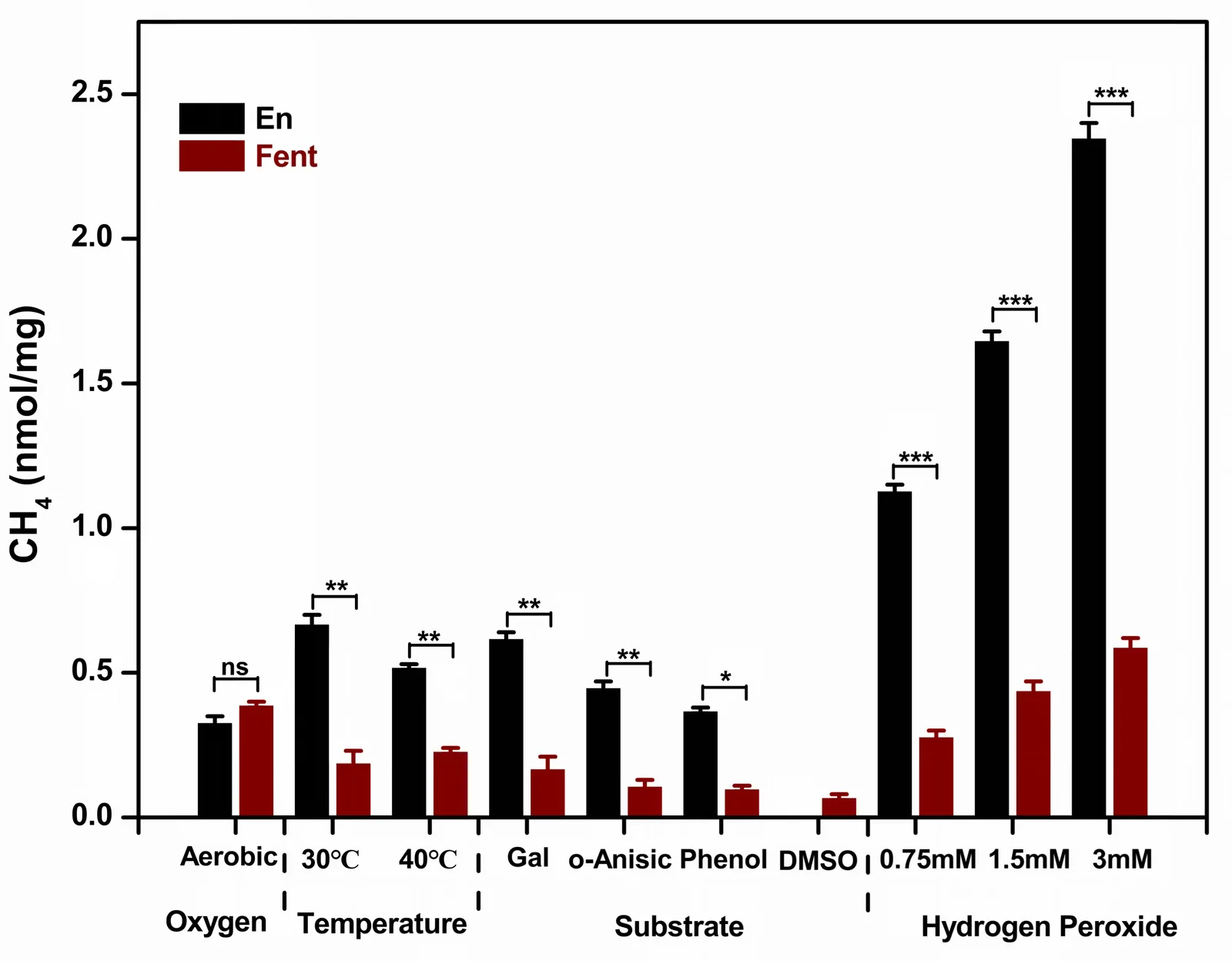
Effect of environmental factors on methane production by *S. commune* via enzymatic and non-enzymatic pathways. *P < 0.05, **P < 0.01, ***P < 0.001; ns, not significant (*P* ≥ 0.05).

The results demonstrated that oxygen exposure exerts opposing regulatory effects on the two methanogenic pathways (Fig. 3). At 30°C under aerobic conditions (21% O_2_), methane production attributable to enzymatic activity was 0.33 nmol/mg, whereas methane yield from iron-dependent non-enzymatic processes was 0.39 nmol/mg, corresponding to an 18.2% higher yield of the non-enzymatic pathway relative to the enzymatic pathway. Conversely, under anaerobic conditions, methane production from the enzymatic activity increased to 0.67 nmol/mg, whereas the yield from the non-enzymatic pathway was only 0.19 nmol/mg, representing a 71.9% lower contribution of the non-enzymatic pathway compared to the enzymatic pathway.

Critically, both the enzymatic and non-enzymatic pathways remain active under both aerobic and anaerobic conditions: oxygen suppresses the enzymatic methanogenesis pathway by ~51% (from 0.67 nmol/mg under anaerobic conditions to 0.33 nmol/mg under aerobic conditions) while activating the iron-dependent non-enzymatic Fenton pathway by ~105% (from 0.19 nmol/mg under anaerobic conditions to 0.39 nmol/mg under aerobic conditions), with no complete shutdown of either pathway under either oxygen regime.

While total methane production from both pathways trended higher under anaerobic (0.86 nmol/mg) than aerobic (0.72 nmol/mg) conditions, an independent sample *t*-test with three biological replicates confirmed that this difference is statistically significant (*P* = 0.012), ruling out the possibility that the ~16% difference was a random artifact. These opposing, pathway-specific responses to oxygen represent differential regulation of *S. commune*’s methanogenic system rather than a complete binary switch between pathways: both pathways contribute to total methane output under all tested oxygen conditions, with only their relative contributions shifting in response to oxygen availability.

This oxygen-induced differential regulation partially compensates for the suppression of the enzymatic methanogenesis pathway, allowing the organism to sustain a substantial fraction of its methanogenic capacity even under aerobic conditions where the enzymatic pathway is inhibited. The Fenton reaction can utilize hydrogen peroxide and other ROS as reactants to support this residual methane production, illustrating the metabolic plasticity of *S. commune* in response to varying environmental oxygen levels (32, 33).

Temperature significantly influences methane production, with higher temperatures adversely affecting the efficiency of the enzymatic pathway. At 40°C, methane yields from the enzymatic pathway dropped to 0.52 nmol/mg, reflecting a 22.4% reduction compared to yields at 30°C. In contrast, methane yields associated with the iron-dependent non-enzymatic process remained stable at 0.18 nmol/mg, indicating that enzymatic processes are more susceptible to thermal stress (Fig. 3). This finding suggests that the enzymes responsible for methane synthesis in *S. commune*, specifically mct and dh, may be inhibited at elevated temperatures, thereby limiting their catalytic functionality. The established temperature optimum for related fungi such as *Laetiporus sulphureus* and *Pleurotus sapidus* concerning non-enzymatic pathways under aerobic conditions is ~27°C, which aligns with our findings (34). In contrast, methanogenic archaea exhibit considerable heat tolerance, maintaining metabolic functions at temperatures exceeding 40°C (35). Adaptations observed in members of the order Archaeoglobales, such as Candidatus Nezhaarchaeota, allow them to switch between metabolic pathways in response to temperature fluctuations, thus sustaining methane production under varying conditions (36). This comparison suggests that fungi may play a more significant role in methane production and emission within the ocean than in the subseafloor biosphere (37).

Substrate type emerged as a critical factor influencing methane production. Carbohydrates, particularly glucose and galactose, yielded the highest outputs, with galactose producing 0.62 nmol/mg for the enzymatic pathway and 0.17 nmol/mg for the Fenton-like pathway. In contrast, aromatic substrates such as 2-methoxybenzoic acid and phenol resulted in significantly lower yields, underscoring the efficiency of carbohydrate metabolism in promoting methane synthesis. When dimethyl sulfoxide (DMSO) served as the sole carbon source, methane production via the enzymatic pathway was completely inhibited, and the Fenton-like pathway’s output decreased to 0.06 nmol/mg (Fig. 3). This substantiates the importance of substrate selection in optimizing methane production mechanisms. The variations in substrate efficiency likely arise from their impact on the availability of methyl precursors. For instance, glucose efficiently converts to methionine, supporting both production pathways (38). In contrast, less efficient substrates such as aromatic compounds necessitate energy-intensive metabolic modifications that limit precursor availability (39). Specialized methyl donors such as DMSO do not support core metabolic processes and can hinder methionine synthesis, impairing both enzymatic and Fenton pathways and ultimately affecting methane yields (6). Therefore, substrate choice critically influences the efficiency and capacity of fungal methanogenesis.

Additionally, exposure to H₂O₂ significantly enhanced methane production across both pathways. Treatment with 1.5 mM H₂O₂ increased methane yields from the enzymatic pathway to 1.65 nmol/mg, approximately 2.5 times higher than the control group, while the Fenton pathway yield rose to 0.44 nmol/mg. The consistent ratio of contributions, around 80% from the enzymatic pathway and 20% from the Fenton-like pathway, suggests that H₂O₂ stimulates metabolic flux toward methane production without disrupting the balance between pathways. Similar responses have been documented in *Halobacterium salinarum* DSM 670 and *Bacillus subtilis*, which also exhibit enhanced methane production under oxidative stress conditions (6, 40). This indicates that methane production could represent an adaptive strategy for fungi facing oxidative environments.

### The role of ROS in methane production in *S. commune*

The exposure of *S. commune* strain 20R-7-F01 to H₂O₂ significantly associated with enhanced methane production capabilities via both enzymatic and non-enzymatic pathways (Fig. 3). It is well established that H₂O₂ exposure elevates intracellular ROS levels, which are reported to influence methane production via non-enzymatic mechanisms (6, 41). We hypothesized that the observed enhancement in methane production under H₂O₂ treatment is linked to increased ROS levels within the fungal mycelium. It is important to note that while the correlational data presented in this study (i.e., significant positive associations between ROS levels and methane yields, quantified as *r* values) support this hypothesis, the current work does not include within-system intervention experiments (e.g., ROS scavenger treatments) to directly test the necessity of ROS as a mediator of H₂O₂-induced methane enhancement. However, our prior study on HHP-induced fungal methane production in deep subseafloor sediments (10) provides direct experimental evidence for this causal role: supplementation with the ROS scavenger butylated hydroxytoluene (BHT) significantly suppressed methane production, confirming that reduced ROS levels are sufficient to inhibit fungal methane yields and supporting the role of ROS as a necessary mediator of enhanced methane production in fungi. To further characterize the relationship between H₂O₂-induced ROS accumulation and methane production in the current study, we measured ROS levels in the fungal mycelia while monitoring methane production across a range of H₂O₂ concentrations (0, 0.75, 1.5, and 3 mM) at 30°C over a 3-day incubation period.

**Fig 4:**
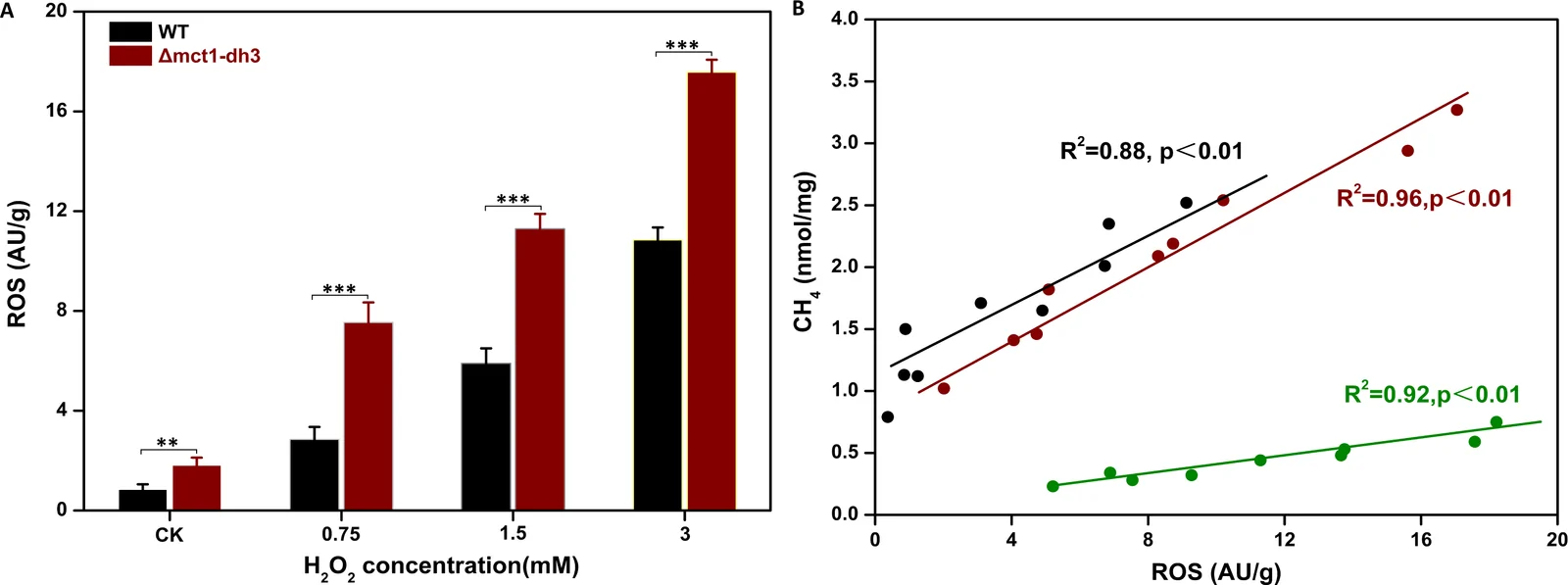
ROS levels (**A**) and their correlation with methane yield (**B**) in *S. commune* exposed to varying H₂O₂ concentrations. Black, red, and green lines indicate correlations with total methane, enzymatic methane, and Fenton reaction methane, respectively. *P < 0.05, **P < 0.01, ***P < 0.001; ns, not significant (*P* ≥ 0.05).

Our results demonstrated a clear dose-dependent increase in ROS levels for both the WT and Δmct1-dh3 strains as H₂O₂ concentrations increased. Specifically, the WT strain exhibited ROS levels of 2.85, 5.9, and 10.85 AU g^−1^, corresponding to H₂O₂ concentrations of 0.75, 1.5, and 3 mM, respectively. Notably, the Δmct1-dh3 strain showed even higher ROS levels, measuring 7.54, 11.29, and 17.57 AU g^−1^ under the same conditions (Fig. 4A). We observed a strong positive correlation between intracellular ROS levels and total methane production (*r* = 0.96), as well as with methane produced via enzymatic (*r* = 0.88) and non-enzymatic (*r* = 0.92) pathways (Fig. 4B). These correlational findings are consistent with the hypothesis that H₂O₂-induced oxidative stress enhances methane formation in *S. commune* strain 20R-7-F01, potentially via elevated ROS levels that facilitate metabolic processes involved in methane synthesis. Furthermore, under 1.5 mM H_2_O_2_ treatment for 3 days, the WT strain showed a methane increment of 0.194 nmol/mg per AU/g ROS, which was significantly higher than the 0.0264 nmol/mg per AU/g observed in the Δmct1-dh3 strain. This result further supports that the enzymatic methanogenesis pathway exhibits a stronger association with ROS levels than the Fenton pathway-dominated Δmct1-dh3 mutant. In addition, we acknowledge that the H₂O₂ concentrations used in this study (0.75–3 mM) are higher than typical environmental levels and may represent an experimental tool for probing pathway mechanisms rather than reflecting natural conditions. Future studies using more environmentally relevant H₂O₂ concentrations and long-term exposure scenarios would be valuable to assess the ecological significance of ROS-mediated methanogenesis.

ROS play multifaceted roles in biological systems, acting as both signaling molecules and intermediates in critical biochemical processes (42). Evidence from our prior work suggests that the enhanced methane production by *S. commune* strain 20R-7-F01 under HHP is associated with elevated ROS levels within the fungal mycelium, which dramatically upregulate the expression of key genes related to methane formation, such as *mct* and *dh* (10). Importantly, the BHT intervention experiments in that study confirmed that ROS are a necessary mediator of this pressure-induced methane enhancement. Additionally, ROS have been reported to activate methyl-coenzyme M reductase in deep-sea hydrogenotrophic methanogenic archaea, leading to increased methane production (43). Concurrently, ROS are associated with enhanced methane production in microorganisms including *Rhodobacter capsulatus* and *Corynebacterium glutamicum* ATCC 13032 via the non-enzymatic Fenton reaction pathway (6). Collectively, these findings indicate that ROS may play a crucial role in regulating dual pathways of methane synthesis in fungi via either enzymatic or non-enzymatic mechanisms.

It should be noted, however, that the present study has two key limitations regarding causal inference for the role of ROS in H_2_O_2_-induced methane production. First, ROS levels were measured using the DCFH-DA assay, which primarily reflects total intracellular oxidative activity rather than specifically detecting hydroxyl radicals (·OH) generated via Fenton chemistry. Indeed, ·OH is notoriously difficult to capture directly in biological systems due to its extremely short half-life (nanoseconds) and high reactivity (44). Consequently, the DCFH-DA fluorescence signal represents a global elevation of oxidative stress rather than serving as direct evidence for Fenton-derived ·OH. Second, and more critically, the current study presents only correlational evidence between ROS levels and methane production (i.e., *r* values) across different H_2_O_2_ treatment conditions and does not include intervention experiments (e.g., ROS scavenger or inducer treatments) within this specific experimental system to directly test whether ROS are a necessary mediator of the observed H_2_O_2_-induced methane enhancement. While our prior published work (10) provided direct evidence for the necessity of ROS in mediating enhanced fungal methane production under high hydrostatic pressure, the causal role of ROS in the H_2_O_2_ treatment context assessed in the current study remains to be fully validated within this specific system. These inherent limitations should be taken into consideration when interpreting the involvement of ROS in the methane production mechanisms observed here. Nonetheless, the consistent suppression of methane production by the ROS scavenger BHT reported in our prior work, together with the strong positive correlation between total ROS levels and methane yields observed in the current study, collectively supports the involvement of ROS-mediated pathways in H_2_O_2_-induced methane production. Future studies employing more specific detection techniques, such as electron paramagnetic resonance (EPR) spectroscopy or ·OH-specific fluorescent probes, are warranted to further delineate the precise contribution of Fenton-derived ·OH to fungal methane production. Additionally, follow-up experiments adding ROS scavengers or antioxidants to the H_2_O_2_ treatment system in the current study are needed to directly verify the necessity of ROS as a mediator in this specific context. Nevertheless, the molecular mechanisms by which ROS exert this regulatory influence in *S. commune* remain to be fully elucidated.

### Conclusion

This study demonstrates that *S. commune* strain 20R-7-F01 possesses dual methanogenic pathways: an enzymatic pathway dependent on mct1 and dh3, and a non-enzymatic Fenton-like reaction. The coexistence of these two mechanisms in a eukaryotic fungus highlights the metabolic versatility of this organism and expands our understanding of biological methane formation beyond the classical archaeal domain.

Our results further reveal that environmental factors, particularly oxygen and oxidative stress, differentially influence the relative contributions of these pathways. These findings suggest that ROS may serve as a key regulatory node linking environmental conditions to metabolic adjustments in fungal methanogenesis. However, the precise molecular mechanisms underlying this regulation warrant further investigation.

This research highlights the complex interplay of ROS in fungal methanogenesis and positions *S. commune* as a valuable model organism for investigating the regulatory mechanisms governing methane production under oxidative stress. The insights gained from this work contribute to our understanding of biological methane formation within the experimental framework of this study. While extrapolation to broader environmental contexts requires caution, these findings may offer preliminary insights into the potential role of fungi in methane cycling under relevant oxidative conditions. Future investigations should focus on elucidating the molecular pathways through which ROS influence methane production, which would provide a more robust foundation for assessing their potential implications in natural ecosystems.

## Data Availability

Data will be made available upon request.

## SUPPLEMENTAL MATERIAL

Supplemental material aem.01298-26-s0001.pdf Tables S1 to S3; Fig. S1 and S2.

ASM does not own the copyrights to Supplemental Material that may be linked to, or accessed through, an article. The authors have granted ASM a non-exclusive, world-wide license to publish the Supplemental Material files. Please contact the corresponding author directly for reuse.

## References

[1] Saunois M, Stavert AR, Poulter B, Bousquet P, Canadell JG, Jackson RB, Raymond PA, Dlugokencky EJ, Houweling S, Patra PK, et al.. 2020. The global methane budget 2000–2017. Earth Syst Sci Data 12:1561–1623. doi:10.5194/essd-12-1561-2020

[2] Keppler F, Hamilton JTG, Brass M, Röckmann T. 2006. Methane emissions from terrestrial plants under aerobic conditions. Nature 439:187–191. doi:10.1038/nature04420 16407949

[3] North JA, Narrowe AB, Xiong W, Byerly KM, Zhao G, Young SJ, Murali S, Wildenthal JA, Cannon WR, Wrighton KC, Hettich RL, Tabita FR. 2020. A nitrogenase-like enzyme system catalyzes methionine, ethylene, and methane biogenesis. Science 369:1094–1098. doi:10.1126/science.abb6310 32855335

[4] Repeta DJ, Ferrón S, Sosa OA, Johnson CG, Repeta LD, Acker M, DeLong EF, Karl DM. 2016. Marine methane paradox explained by bacterial degradation of dissolved organic matter. Nature Geosci 9:884–887. doi:10.1038/ngeo2837

[5] Enami S, Sakamoto Y, Colussi AJ. 2014. Fenton chemistry at aqueous interfaces. Proc Natl Acad Sci USA 111:623–628. doi:10.1073/pnas.1314885111 PMC3896178 24379389

[6] Ernst L, Steinfeld B, Barayeu U, Klintzsch T, Kurth M, Grimm D, Dick TP, Rebelein JG, Bischofs IB, Keppler F. 2022. Methane formation driven by reactive oxygen species across all living organisms. Nature 603:482–487. doi:10.1038/s41586-022-04511-9 35264795

[7] Lenhart K, Bunge M, Ratering S, Neu TR, Schüttmann I, Greule M, Kammann C, Schnell S, Müller C, Zorn H, Keppler F. 2012. Evidence for methane production by saprotrophic fungi. Nat Commun 3:1046. doi:10.1038/ncomms2049 22948828

[8] Colosimo F, Thomas R, Lloyd JR, Taylor KG, Boothman C, Smith AD, Lord R, Kalin RM. 2016. Biogenic methane in shale gas and coal bed methane: a review of current knowledge and gaps. Int J Coal Geol 165:106–120. doi:10.1016/j.coal.2016.08.011

[9] Huang X, Liu X, Xue Y, Pan B, Xiao L, Wang S, Lever MA, Hinrichs KU, Inagaki F, Liu C. 2022. Methane production by facultative anaerobic wood-rot fungi via a new halomethane-dependent pathway. Microbiol Spectr 10:e01700-22. doi:10.1128/spectrum.01700-22 PMC9604129 36102652

[10] Zhao M, Li D, Liu J, Fang J, Liu C. 2024. Fungal methane production under high hydrostatic pressure in deep subseafloor sediments. Microorganisms 12:2160. doi:10.3390/microorganisms12112160 PMC11596643 39597547

[11] Mukhin VA, Voronin PYu. 2007. Methane emission during wood fungal decomposition. Dokl Biol Sci 413:159–160. doi:10.1134/S0012496607020202

[12] Harper DB, Buswell JA, Kennedy JT, Hamilton JT. 1990. Chloromethane, methyl donor in veratryl alcohol biosynthesis in Phanerochaete chrysosporium and other lignin-degrading fungi. Appl Environ Microbiol 56:3450–3457. doi:10.1128/aem.56.11.3450-3457.1990 PMC184980 16348350

[13] Opara A, Adams DJ, Free ML, McLennan J, Hamilton J. 2012. Microbial production of methane and carbon dioxide from lignite, bituminous coal, and coal waste materials. Int J Coal Geol 96–97:1–8. doi:10.1016/j.coal.2012.02.010

[14] Zhou S, Fushinobu S, Kim S-W, Nakanishi Y, Wakagi T, Shoun H. 2010. *Aspergillus oryzae* flavohemoglobins promote oxidative damage by hydrogen peroxide. Biochem Biophys Res Commun 394:558–561. doi:10.1016/j.bbrc.2010.03.018 20211603

[15] Ottow JC, Von Klopotek A. 1969. Enzymatic reduction of iron oxide by fungi. Appl Microbiol 18:41–43. doi:10.1128/am.18.1.41-43.1969 PMC377882 16349855

[16] Naito Y, Hino K, Bono H, Ui-Tei K. 2015. CRISPRdirect: software for designing CRISPR/Cas guide RNA with reduced off-target sites. Bioinformatics 31:1120–1123. doi:10.1093/bioinformatics/btu743 PMC4382898 25414360

[17] Schuster M, Kahmann R. 2019. CRISPR-Cas9 genome editing approaches in filamentous fungi and oomycetes. Fungal Genet Biol 130:43–53. doi:10.1016/j.fgb.2019.04.016 31048007

[18] Galaris D, Barbouti A, Pantopoulos K. 2019. Iron homeostasis and oxidative stress: an intimate relationship. Biochim Biophys Acta Mol Cell Res 1866:118535. doi:10.1016/j.bbamcr.2019.118535 31446062

[19] Botella C, Hernandez JE, Webb C. 2019. Dry weight model, capacitance and metabolic data as indicators of fungal biomass growth in solid state fermentation. Food Bioprod Process 114:144–153. doi:10.1016/j.fbp.2018.12.002

[20] Basu A, Ray S, Chowdhury S, Sarkar A, Mandal DP, Bhattacharjee S, Kundu S. 2018. Evaluating the antimicrobial, apoptotic, and cancer cell gene delivery properties of protein-capped gold nanoparticles synthesized from the edible mycorrhizal fungus *Tricholoma crassum*. Nanoscale Res Lett 13:154. doi:10.1186/s11671-018-2561-y PMC5955874 29767296

[21] Aldridge JT, Catlett JL, Smith ML, Buan NR. 2016. Methods for detecting microbial methane production and consumption by gas chromatography. Bio Protoc 6:e1779. doi:10.21769/bioprotoc.1779 PMC4993457 27559541

[22] Chen MF, Li YJ, Yang TL, Lou B, Xie XM. 2009. Losartan inhibits monocytic adhesion induced by ADMA via downregulation of chemokine receptors in monocytes. Eur J Clin Pharmacol 65:457–464. doi:10.1007/s00228-008-0607-2 19156404

[23] Qian H, Chen W, Li J, Wang J, Zhou Z, Liu W, Fu Z. 2009. The effect of exogenous nitric oxide on alleviating herbicide damage in *Chlorella vulgaris*. Aquat Toxicol 92:250–257. doi:10.1016/j.aquatox.2009.02.008 19297032

[24] Livak KJ, Schmittgen TD. 2001. Analysis of relative gene expression data using real-time quantitative PCR and the 2^−ΔΔ^*^C^*^_T_^ method. Methods 25:402–408. doi:10.1006/meth.2001.1262 11846609

[25] Einav S, O’Connor M. 2019. P-values and significance: the null hypothesis that they are not related is correct. J Crit Care 54:159–162. doi:10.1016/j.jcrc.2019.08.020 31472396

[26] Cordente AG, Heinrich A, Pretorius IS, Swiegers JH. 2009. Isolation of sulfite reductase variants of a commercial wine yeast with significantly reduced hydrogen sulfide production. FEMS Yeast Res 9:446–459. doi:10.1111/j.1567-1364.2009.00489.x 19236486

[27] Levati E, Sartini S, Bolchi A, Ottonello S, Montanini B. 2016. Moonlighting transcriptional activation function of a fungal sulfur metabolism enzyme. Sci Rep 6:25165. doi:10.1038/srep25165 PMC4848566 27121330

[28] Cui R, Liu S, Wang C, Liu T, Ren J, Jia Y, Tong Y, Liu C, Zhang J. 2020. Methane-rich saline alleviates CA/CPR brain injury by inhibiting oxidative stress, microglial activation-induced inflammatory responses, and er stress-mediated apoptosis. Oxid Med Cell Longev 2020:1–13. doi:10.1155/2020/8829328 PMC7603629 33149813

[29] Samma MK, Zhou H, Cui W, Zhu K, Zhang J, Shen W. 2017. Methane alleviates copper-induced seed germination inhibition and oxidative stress in *Medicago sativa*. Biometals 30:97–111. doi:10.1007/s10534-017-9989-x 28091954

[30] Wishkerman A, Greiner S, Ghyczy M, Boros M, Rausch T, Lenhart K, Keppler F. 2011. Enhanced formation of methane in plant cell cultures by inhibition of cytochrome c oxidase. Plant Cell Environ 34:457–464. doi:10.1111/j.1365-3040.2010.02255.x 21062320

[31] Dong Y, Qi L, Zhao F, Chen Y, Liang L, Wang J, Zhao W, Wang F, Xu H. 2025. Uncovering dynamic transcriptional regulation of methanogenesis via single-cell imaging of archaeal gene expression. Nat Commun 16:2255. doi:10.1038/s41467-025-57159-0 PMC11885431 40050284

[32] Jiang JP, Leng S, Liao YF, Liu X, Li DX, Chu C, Yu XY, Liu CH. 2023. The potential role of subseafloor fungi in driving the biogeochemical cycle of nitrogen under anaerobic conditions. Sci Total Environ 897:165374. doi:10.1016/j.scitotenv.2023.165374 37422230

[33] Zain Ul Arifeen M, Ahmad S, Wu X, Hou S, Liu C. 2024. Amino acids could sustain fungal life in the energy-limited anaerobic sediments below the seafloor. Appl Environ Microbiol 90:e0127924. doi:10.1128/aem.01279-24 PMC11497798 39302086

[34] Schroll M, Lenhart K, Bender T, Hötten P, Rudolph A, Sörensen S, Keppler F. 2024. Fungal methane production controlled by oxygen levels and temperature. Methane 3:257–275. doi:10.3390/methane3020015

[35] Ginsbach LF, Gonzalez JM. 2022. Understanding Life at high temperatures: relationships of molecular channels in enzymes of methanogenic archaea and their growth temperatures. Int J Mol Sci 23:15149. doi:10.3390/ijms232315149 PMC9741079 36499474

[36] Wang J, Qu YN, Evans PN, Guo Q, Zhou F, Nie M, Jin Q, Zhang Y, Zhai X, Zhou M, Yu Z, Fu QL, Xie YG, Hedlund BP, Li WJ, Hua ZS, Wang Z, Wang Y. 2023. Evidence for nontraditional *mcr*-containing archaea contributing to biological methanogenesis in geothermal springs. Sci Adv 9:eadg6004. doi:10.1126/sciadv.adg6004 PMC10306296 37379385

[37] Ivarsson M, Bengtson S, Drake H, Francis W. 2018. Fungi in deep subsurface environments. Adv Appl Microbiol 102:83–116. doi:10.1016/bs.aambs.2017.11.001 29680127

[38] Cai M, Liu Z, Zhao Z, Wu H, Xu M, Rao Z. 2023. Microbial production of L-methionine and its precursors using systems metabolic engineering. Biotechnol Adv 69:108260. doi:10.1016/j.biotechadv.2023.108260 37739275

[39] Bond DR, Holmes DE, Tender LM, Lovley DR. 2002. Electrode-reducing microorganisms that harvest energy from marine sediments. Science 295:483–485. doi:10.1126/science.1066771 11799240

[40] Horne AJ, Lessner DJ. 2013. Assessment of the oxidant tolerance of *Methanosarcina acetivorans*. FEMS Microbiol Lett 343:13–19. doi:10.1111/1574-6968.12115 PMC3651780 23448147

[41] Lennicke C, Cochemé HM. 2021. Redox metabolism: ROS as specific molecular regulators of cell signaling and function. Mol Cell 81:3691–3707. doi:10.1016/j.molcel.2021.08.018 34547234

[42] Hong Y, Boiti A, Vallone D, Foulkes NS. 2024. Reactive oxygen species signaling and oxidative stress: transcriptional regulation and evolution. Antioxidants (Basel) 13:312. doi:10.3390/antiox13030312 PMC10967436 38539845

[43] Mauerhofer L-M, Zwirtmayr S, Pappenreiter P, Bernacchi S, Seifert AH, Reischl B, Schmider T, Taubner R-S, Paulik C, Rittmann SK-MR. 2021. Hyperthermophilic methanogenic archaea act as high-pressure CH_4_ cell factories. Commun Biol 4:289. doi:10.1038/s42003-021-01828-5 PMC7935968 33674723

[44] Winterbourn CC. 2014. The challenges of using fluorescent probes to detect and quantify specific reactive oxygen species in living cells. Biochim Biophys Acta 1840:730–738. doi:10.1016/j.bbagen.2013.05.004 23665586

