# Supplementary material for: A dual methane production mechanism mediated by enzymatic catalysis and the Fenton reaction in the wood-rotting fungus Schizophyllum commune
Source: Appl Environ Microbiol. 2026 Aug 27;92(9):e01298-26. doi: 10.1128/aem.01298-26 (PMC13599528; doi:10.1128/aem.01298-26)
Supplement: Supplementary file 1 [file aem.01298-26-s0001.pdf]

Supplementary Materials for

A Dual Methane Production Mechanism Mediated by Enzymatic Catalysis and Fenton Reaction in the Wood-Rotting Fungus *Schizophyllum commune*

Mengshi Zhao[a], Yu Xiao[b], Shuang Leng[a], Jiasong Fang[b,c], Changhong Liu[a,]*

[a]State Key Laboratory of Pharmaceutical Biotechnology, Nanjing University, Nanjing 210023, China

[b]Shanghai Engineering Research Center of Hadal Science and Technology, College of Marine Sciences, Shanghai Ocean University, Shanghai 201306, PR China

[c]Laboratory for Marine Biology and Biotechnology, Qingdao Marine Science and Technology Center, Qingdao 266071, China.

∗Correspondence: chliu@nju.edu.cn

Table S1 SgRNA/Primers

SgRNA/Primer	Sequence (5'-3')
mct1-SgRNA1	GGGATATTGCATGGTGCGTG
mct1-SgRNA2	AAGGTCGTTCCTTGGCGTAT
dh3-SgRNA1	AATACTACGACCACCTCCCG
dh3-SgRNA2	GCCTTATCTCATTCGCACTT
Ko-mct1-F	ATGGAGGAAACACCGAGGACG
Ko-mct1-R	GATGGTTTGGCGGAGGAAGTAG
Ko-dh3-F	ACCCCGCCTACCACCAATACTA
Ko-dh3-R	TTACACGCCGACCAATTCCTTC
LentiCRISPRv2-F	TAGTAGACATAATAGCAACAG
LentiCRISPRv2-R	GCCAGTACACGACATCACTT
pCAMBIA1302-F	ATCTGTCACTTTATTGTGAA
pCAMBIA1302-R	TTTCCAGTAGTGCAAATAAA

Note:KO-primer is a Mutant Allele-Specific Primer; LentiCRISPRv2-primer is a CRISPR Knockout Vector Construction Verification Primer;pCAMBIA1302-primer is a Rescue/Complementation Expression Vector Construction Verification Primer.

Table S2 RT-qPCR primers

Primer ID	Sequence (5'-3')
mct1-F	ATTCCTGGGATATTGCATGG
mct1-R	GTCGAATATGCGAGTTAGCTC
dh3-F	TCACTCCCCAGCCCAACC
dh3-R	GCTTGTCCGTCTCGCCG
action-F	GTCCGCCCTCGAGAAGAGTTA
action-R	TTGTACGTCGTCTCGTGGATA

Table S3 Methane yield comparison

CH_4(nmol/mg) / Time(d)	A	B	C	B+C	A-B	A-C
1	0.67±0.03^{a}	0.55±0.01^{b}	0.17±0.03^{c}	0.72±0.03^{a}	0.12±0.03^{c}	0.50±0.04^{b}
3	0.81±0.04^{d}	0.67±0.03^{a}	0.19±0.04^{c}	0.86±0.05^{d}	0.14±0.05^{c}	0.62±0.06^{a}
5	1.05±0.07^{e}	0.89±0.04^{d}	0.20±0.03^{c}	1.09±0.05^{e}	0.16±0.08^{c}	0.85±0.08^{d}

Note:Data are shown as mean ±SD. A: WT (iron-replete); B: WT (iron-depleted); C: Δ mct1-dh3 (iron-replete). Different letters indicate significant differences (*p* < 0.05); same or no letters indicate no significant difference (*p*≥0.05).

Figure S1 Genetic characterization of the mutant strains

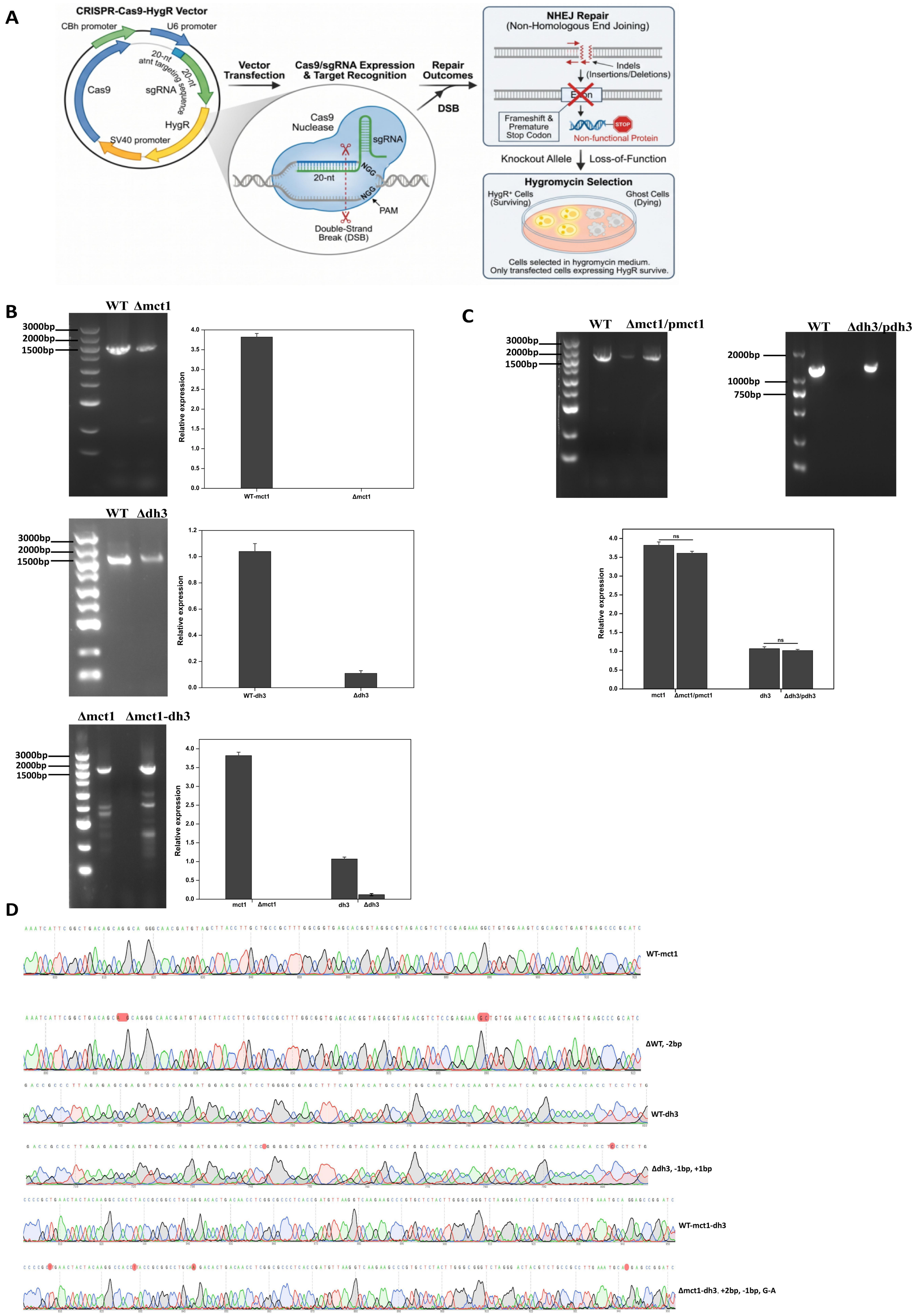

Fig. S1. A: Schematic representation of the gene knockout strategy. B: PCR validation of gene deletion and relative transcriptional expression levels of Δmct1, Δdh3, and Δmct1-Δdh3 strains compared to the Wild-Type (WT) strain. C:PCR validation and

relative transcriptional expression levels of the complementation strains Δmct1/pmct1 and Δdh3/pdh3 compared to the WT strain. D: Sequencing confirmation of the nucleotide deletion mutations in Δmct1, Δdh3, and Δmct1-Δdh3 strains.

Figure S2

A:0.1MPa-30°C

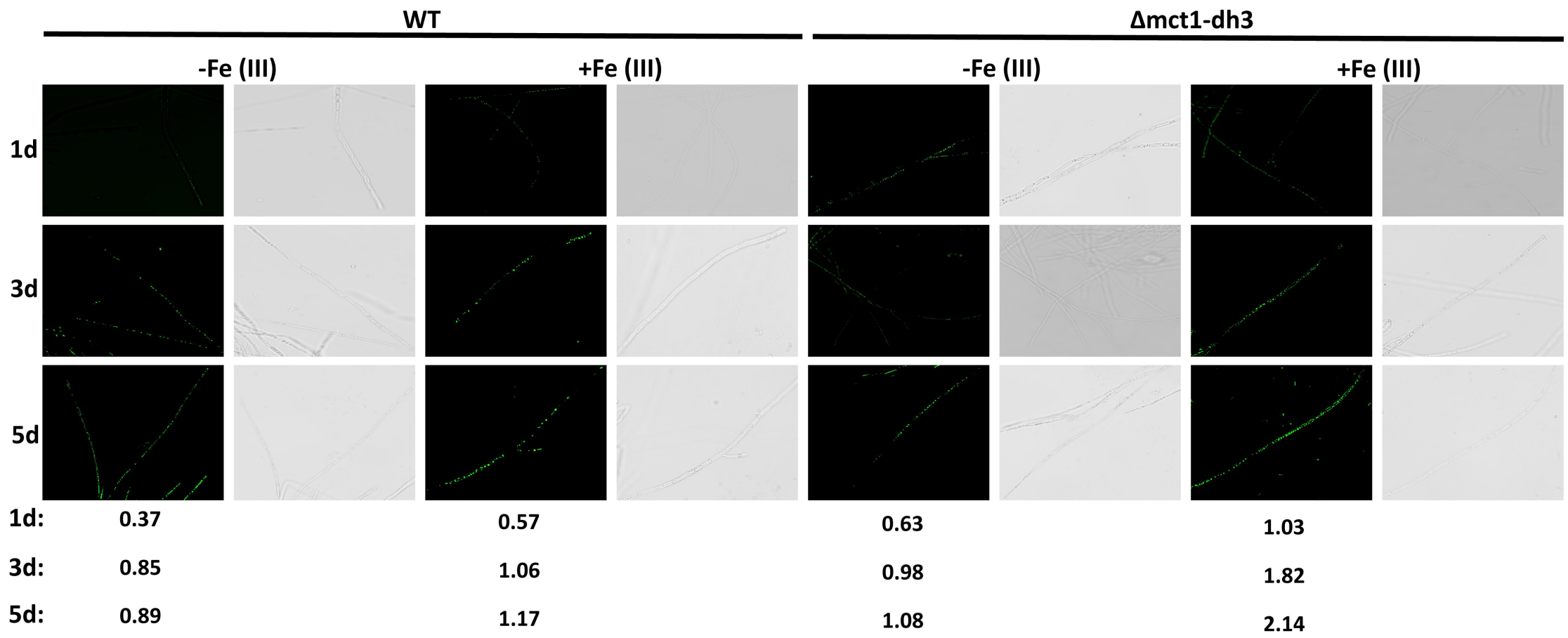

B：0.1MPa-40°C

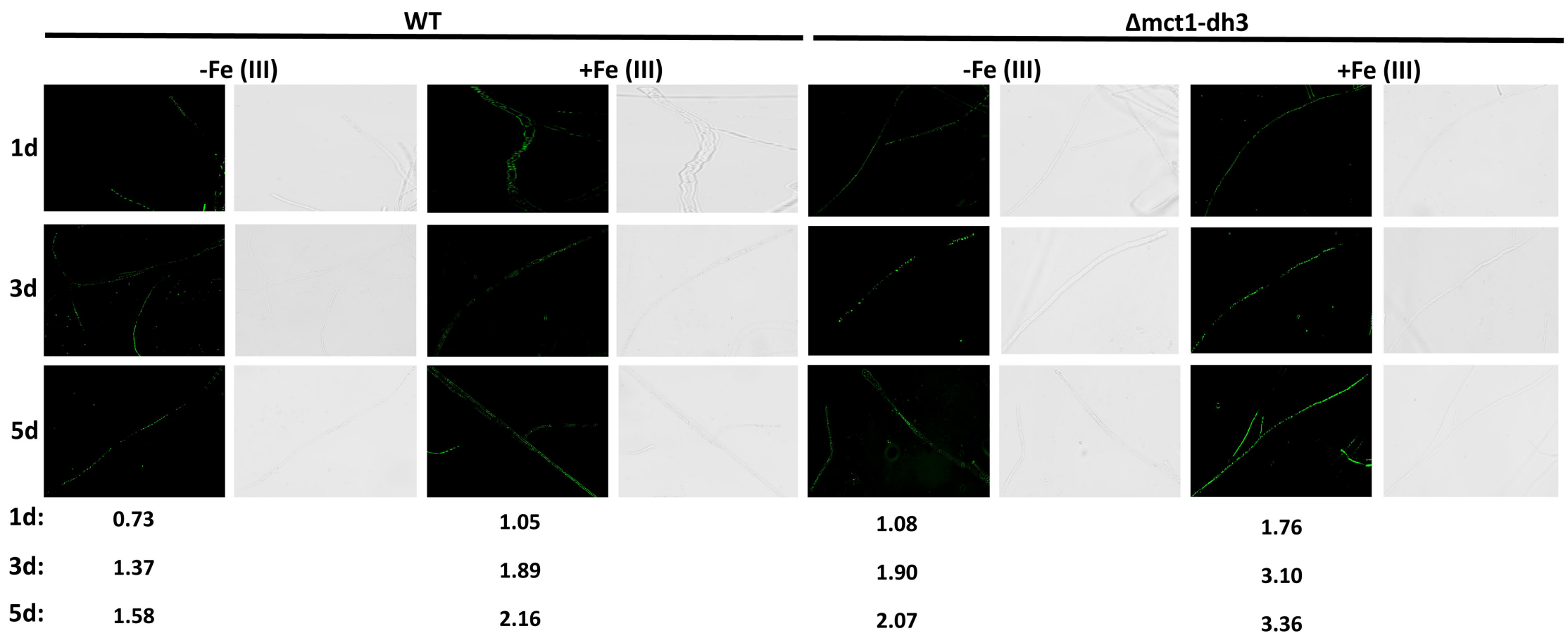

C：Gal

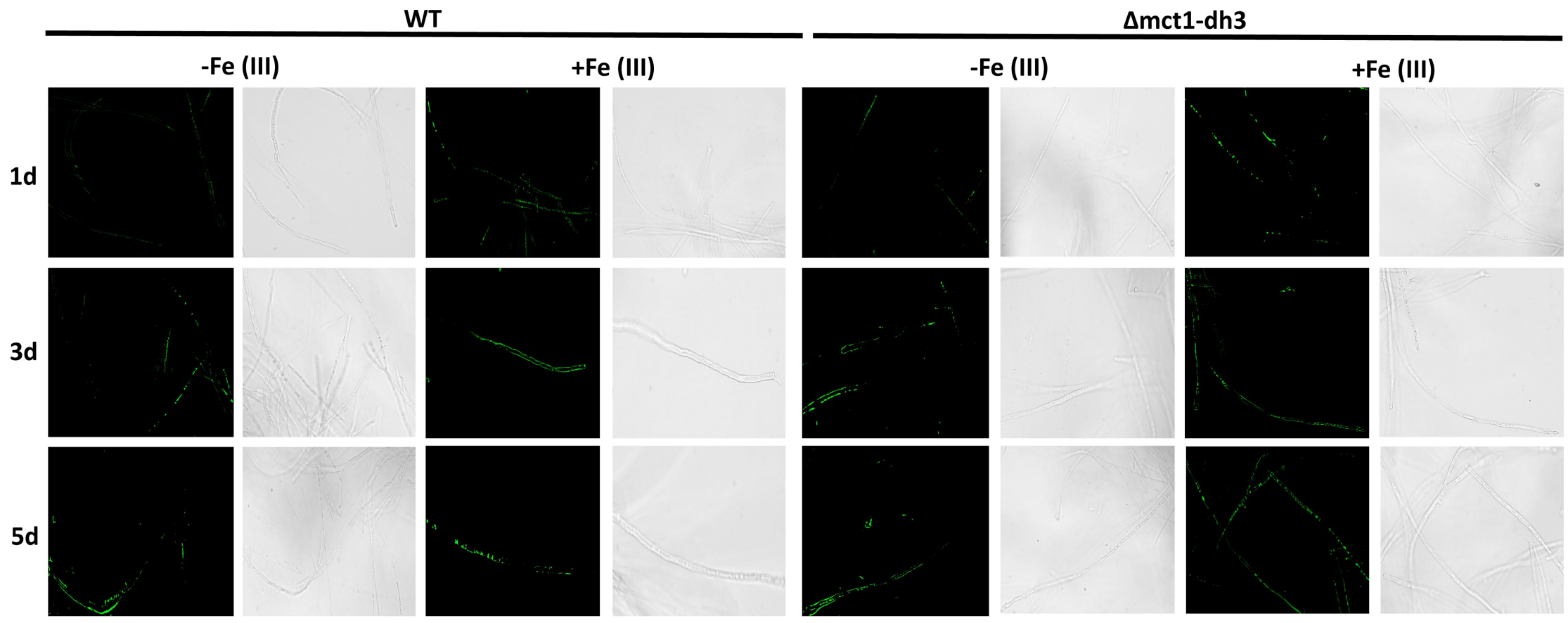

1d:	0.32	0.50	0.53	0.95
3d:	0.79	0.89	0.89	1.53
5d:	0.82	1.04	0.94	1.93

D:o-Anisic acid

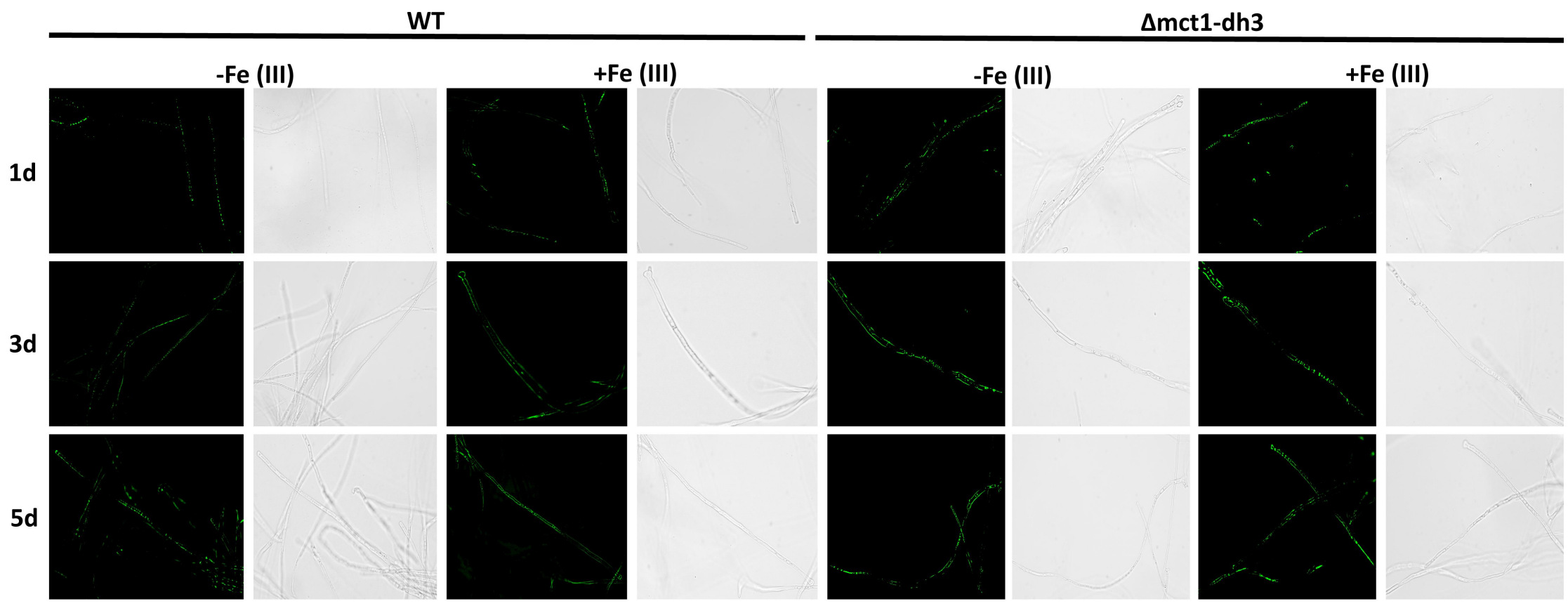

1d:	0.47	0.62	0.68	1.15
3d:	0.95	1.16	1.08	1.94
5d:	0.99	1.21	1.17	2.25

E: Phenol

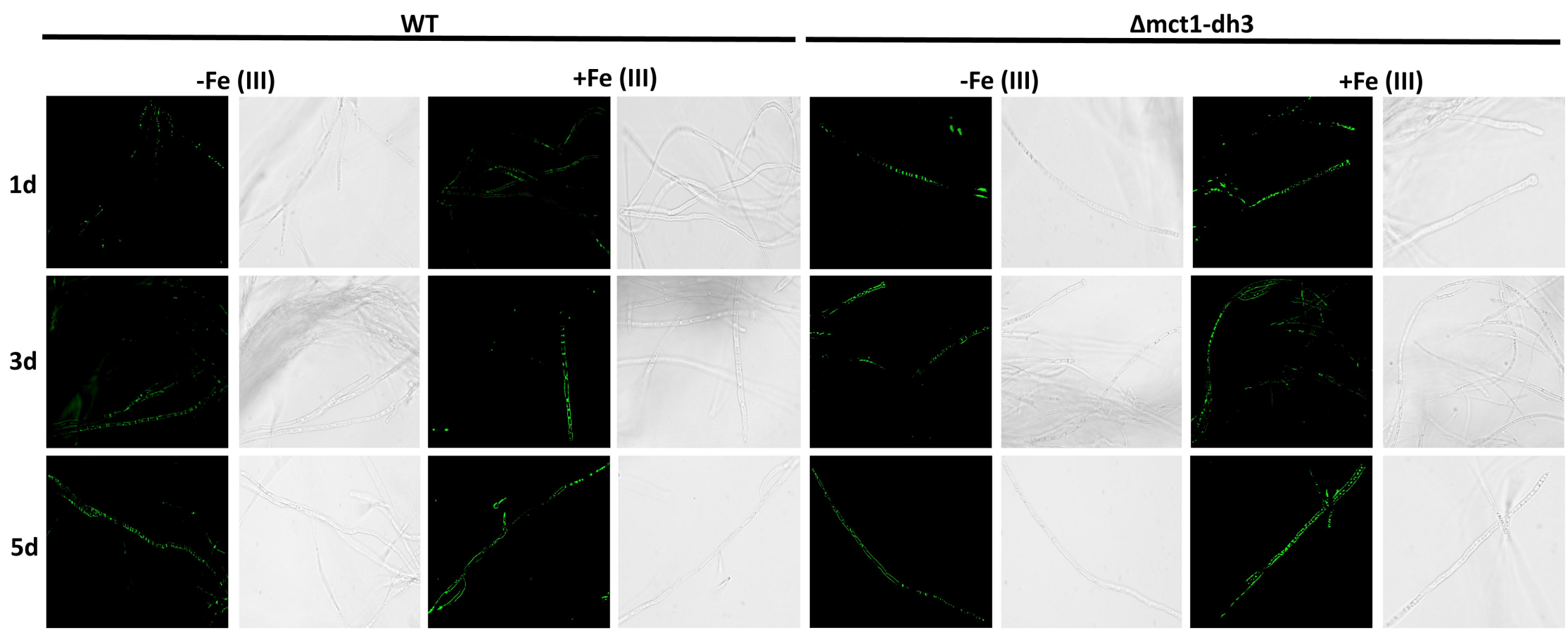

1d:	0.51	0.67	0.75	1.22
3d:	1.00	1.23	1.16	2.03
5d:	1.05	1.29	1.23	2.36

F: DMSO

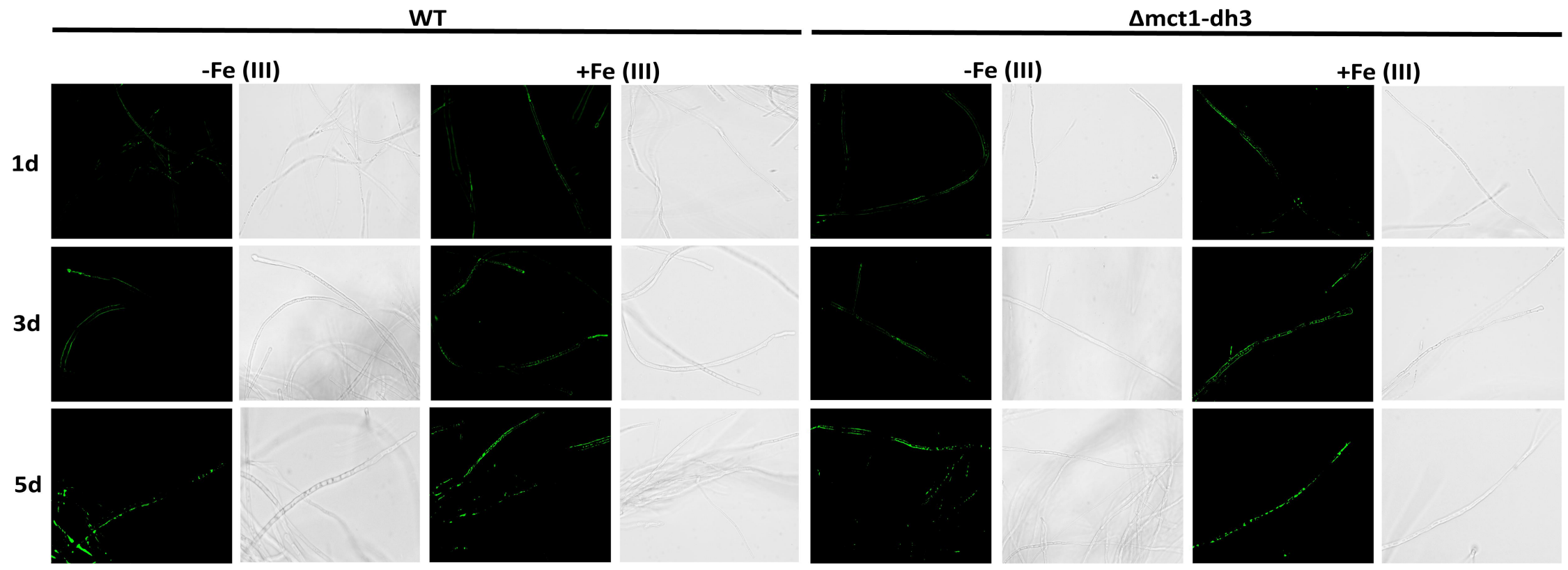

1d:	0.34	0.53	0.62	1.06
3d:	0.84	1.03	0.97	1.79
5d:	0.88	1.16	1.09	2.10

G： 0.75mM-H2O2

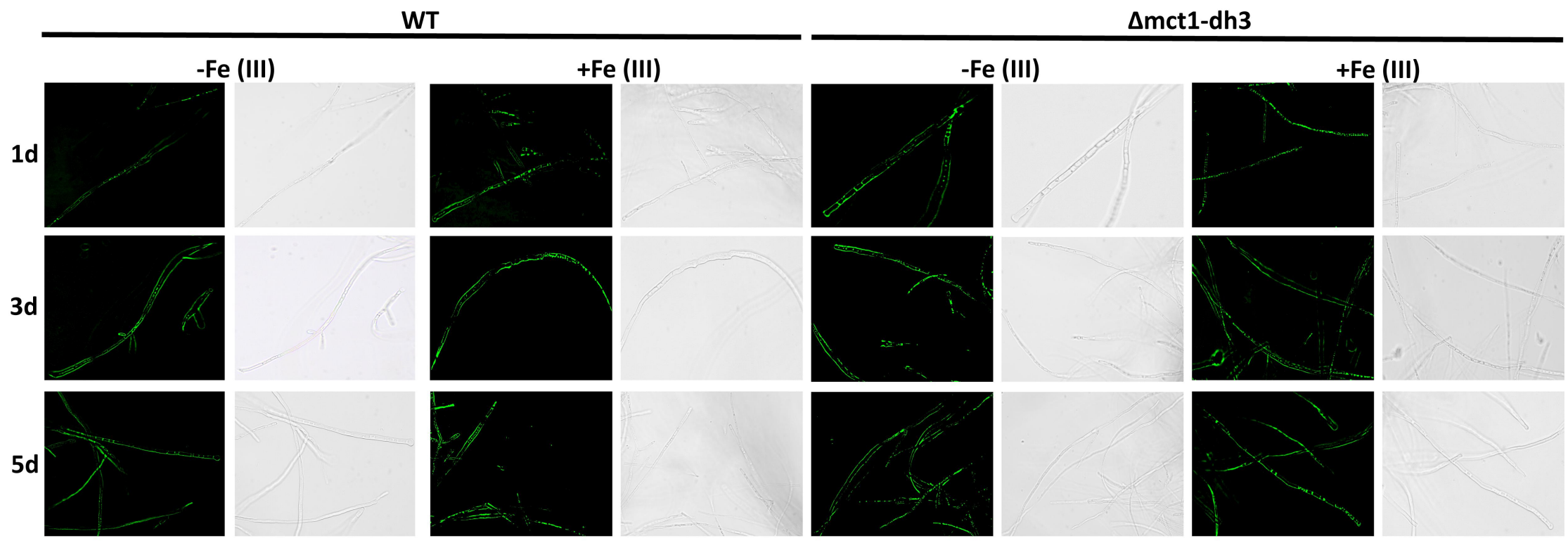

1d:	1.21	2.02	3.28	5.21
3d:	2.85	4.06	4.83	7.54
5d:	3.35	5.09	5.67	9.27

H： 1.5mM-H_2O_2

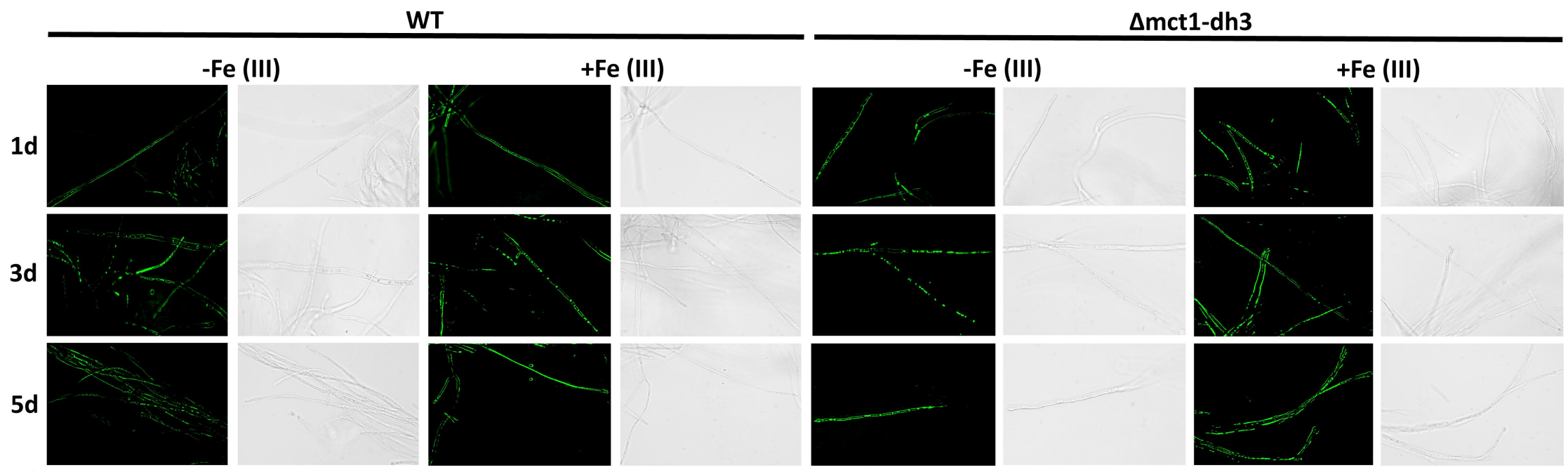

1d:	2.51	4.73	5.09	6.89
3d:	5.90	8.29	8.20	11.29
5d:	7.40	10.20	9.75	13.76

I： 3mM-H_2O_2

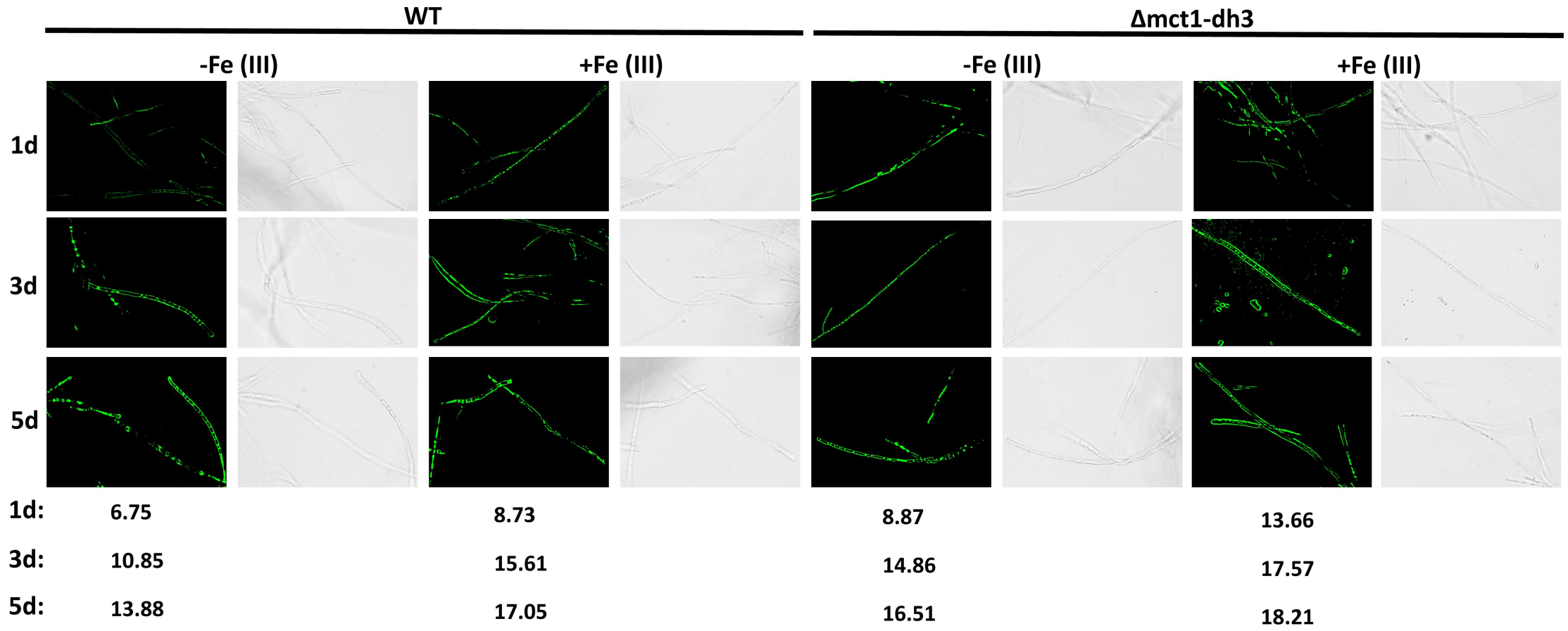

Fig. S2. A-I: Intracellular ROS in wild-type versus Δmct1-dh3 mutant strains upon exposure to different environmental stressors.
